# The Early History of the Hospital Sunday and Saturday Funds

**Published:** 1894-09-01

**Authors:** 


### The Institutional Workshop.

### THE EARLY HISTORY OF THE HOSPITAL SUNDAY AND SATURDAY FUNDS.

### HOSPITAL SUNDAY.

Periodically claims spring up on the part of individuals who seek for themselves or a friend honour or credit, or both, on account of their being the founders, or originators, or suggestors of the idea, or the name of Hospital Sunday or Hospital Saturday. Thus in 1880 an anonymous correspondent wrote to the London papers to declare:—

It is not correct to state that the late Canon Miller was the founder of Hospital Sunday. What he did twenty-five years ago, when he was rector of Birmingham, was to start in that town what were then and are still called "Periodical Collections for Local Charities." The Hospital Sunday was first established in Manchester in 1870, and was the work of a well-known gentleman, the Rev. John Henn, the rector of St. John's Church. The movement proving very successful was at once copied by the Liverpool people, and afterwards by other towns throughout the country, the plan of our Hospital Sunday scheme, with slight variations, being in all cases adopted. In due course London followed suit at the suggestion of some gentlemen, who thought that what had proved so successful in Manchester and Liverpool and elsewhere might succeed there too.

Despite the precise refutation set up in 1880 in regard to this claim in favour of the Rev. John Henn, whose services to the medical charities of Manchester we fully recognise and appreciate, in 1884 Mr. George Huntingdon produced what he called indisputable evidence that the Rev. John Henn was the originator of Hospital Sunday, namely, the presentation of a piece of plate in May, 1873, "in recognition of his services for upwards of four years as secretary of the Manchester and Salford Hospital Sunday Movement." "Therefore," concluded Mr. Huntingdon, as the Rev. J. W. Marshall pointed out at the time, "Mr. Henn's services must have been called into requisition early in 1869." The Rev. J. W. Marshall then proceeds:—

I have before me the Birmingham Red Book. On page 43 it appears that the first Hospital Sunday in that town occurred in 1859, just ten years before Mr. Henn's services were called into requisition. I have yet to learn that in any other town the idea of having collections on one Sunday at all the places of worship on behalf of hospitals had been carried out prior to 1859.

Since then this claim has not been reiterated, but in *Forward* for August, 1894, page 75, appears a letter from the Rev. John Henn, dated June 27th last, in the course of which he states:—

Birmingham originated neither Hospital Sunday nor Hospital Saturday, so called. In 1860 Canon Miller started the simultaneous movement known, I believe, to themselves as "Periodical Collections for Local Charities," at least one of which is not a hospital in the ordinary sense; but Birmingham at once used our title when it appeared. If I am wrong, no doubt you can correct me.

The point in question is this: Did Birmingham ever use the title of Hospital Sunday before 1870?

The answer to this question was supplied by Mr. Marshall's reference to the Birmingham Red Book published in the *Standard*, June 11th, 1884. The columns of the Birmingham daily papers prior to 1870 would be further evidence, and the columns of the *Lancet* prior to the same year, in which the late Dr. Wakley wrote a number of articles on Hospital Sunday, prove that the title, "Hospital Sunday," was used before 1870, and generally accepted, not only in Birmingham, but by the press, as describing the collections in Birmingham.

The answer then to Mr. Henn's question is that Birmingham did undoubtedly use the title of Hospital Sunday before 1870, and the present writer, as an old resident in Birmingham from 1865 onwards, remembers that the collections were always popularly known as Hospital Sunday, and that many residents made a point of going to church on that day for that reason, whatever they might do on other Sundays throughout the year. But the best reply which can be given to the Manchester claim is a letter dated July 19th, 1875, which the Rev. John Henn addressed to Mr. Burdett, and which lies before us as we write. The material part of it is as follows:—

If you are thinking of starting a Hospital Sunday you cannot do better than consult Canon Miller, who began what he called "Periodical Collections for Local Charities" in Birmingham in 1858. It was on the model of this I started our Hospital Sunday Fund here, and all the rest of the Hospital Sundays are copies of ours.

Anyone who is interested in this subject should refer to chapter 10, volume 3, of "Hospitals and Asylums of the World" (Scientific Press), from which we quote the following:—

This extract from Mr. Henn's letter is pretty conclusive, but we would add to it the fact that when Hospital Sunday —a name always popularly used in Birmingham to describe these collections—was first mooted in London, the then Mayor of Birmingham, Mr. Alderman Biggs, brought the author a letter from Sir Sydney Waterlow, asking him to obtain full particulars of the Hospital Sunday organisation in Birmingham, and that thereupon he collected the facts asked for, and Mr. Biggs forwarded them in due course to the Mansion House. Dr. Wakley, editor of the *Lancet*, by repeatedly urging upon the Lord Mayor and the London clergy the duty of organising a Metropolitan Hospital Sunday Fund, originated the movement in London, and to him, more than to any one else, is due the credit of having introduced Hospital Sunday to this metropolis. The origin of Hospital Sunday is, therefore, narrowed to Birmingham.

We may add that Mr. Thomas Barber Wright, a former co-proprietor of the *Midland Counties Herald*, first suggested annual collections in all places of worship; but the late Rev. Canon Miller was in reality the founder of Hospital Sunday in Birmingham, as he devoted much time and great ability to the elaboration and successful working of the movement which is now known as Hospital Sunday, and which has spread not only in this country, but to nearly every English-speaking population throughout the world. In fact, it has become as popular and successful in New York as in London, and the large Australian cities seem to show greater enthusiasm and to collect larger sums than those yet realised in America or in any British cities.

### HOSPITAL SATURDAY.

The letter of the Rev. John Henn, in *Forward*, already referred to, confirms the view expressed in "Hospitals and Asylums of the World" as to the foundation of Hospital Saturday. He states:—

Hospital Sunday in Manchester, as we called it, took the fancy first of Liverpool, our neighbour, and they started their Hospital Sunday in 1871, which they followed by what they called Hospital Saturday, and in 1872 we had our Hospital Saturday.

It is beyond dispute that the title, "Hospital Saturday," originated in Liverpool, but the incongruity of the title, in view of the system and success of the Hospital Saturday in Birmingham, will be apparent from the following quotation, which we take from a pamphlet entitled, "Hospital Sunday—Review of the Movement in Liverpool," dated Jandary 1[illegible]th, 1877. On page 5 of this pamphlet the following paragraph appears :—

1st. Hospital Saturday.—A desire was expressed by some operatives that they should be allowed to contribute in a special way to the Hospital Sunday Fund. Consequently, boxes were prepared by the committee, and these were given to those firms applying for them, as well as given to a few well-known employers of labour. No great effort was made, however, and only some 80 or 90 boxes were distributed; with the boxes handbills were circulated, and a digest of the reports of the selected charities. Some 73 boxes were found to contain £103 13s. 10d., which prompted the committee to increased efforts in this direction in the following years.

At first, and for some years afterwards, Hospital Saturday in Liverpool was managed by the Hospital Sunday Fund Committee, but it is worked at the present time by a special Hospital Saturday Fund Committee, and shows an upward tendency, £4,439 having been collected in 1892, against £4,074 received in 1890.

We have not space to pursue this interesting question in detail on the present occasion, but the facts are stated at length in volume 3 of "Hospitals and Asylums of the World," pp. 193, 194. The author sums up the evidence as follows :—

It will be seen, therefore, that if Liverpool originated Hospital Saturday, to enable the Hospital Sunday Fund Committee to sweep into their net contributions from the working classes which would not otherwise have been obtained for the support of the hospitals, Manchester and Birmingham were speedily pursuing the same course, and that in the case of the latter town the contributions of the working classes had been attracted to the hospitals so early as 1869.

The history of the Birmingham Hospital Saturday Fund has been so fully and recently told in these columns, that it is not necessary to add anything further on the present occasion. The history of Hospital Sunday and Saturday is exhaustively written in "Hospitals and Asylums of the World," and to that book we must refer the reader. In dismissing the subject we desire to recognise fully the services which we the Rev. John Henn has rendered to the Hospital Sunday and Saturday Funds in Manchester; services which have been gratefully and deservedly recognised by his fellow-townsmen. Every impartial person will wish to do full justice to the labours of every one who has contributed in any way to the successful establishment and progress of these funds. Historical accuracy is, however, essential in the interests of all, and that is why we have thought it necessary to place all the main facts on record in this article.

